# A New Front in Microbial Warfare—Delivery of Antifungal Effectors by the Type VI Secretion System

**DOI:** 10.3390/jof5020050

**Published:** 2019-06-14

**Authors:** Katharina Trunk, Sarah J. Coulthurst, Janet Quinn

**Affiliations:** 1 Institute for Cell and Molecular Biosciences, Faculty of Medicine, Newcastle University, Newcastle upon Tyne NE2 4HH, UK; katharina.trunk@ncl.ac.uk; 2 Division of Molecular Microbiology, School of Life Sciences, University of Dundee, Dundee DD1 5EH, UK

**Keywords:** Type VI secretion system, polymicrobial interactions, antifungal effectors

## Abstract

Microbes typically exist in mixed communities and display complex synergistic and antagonistic interactions. The Type VI secretion system (T6SS) is widespread in Gram-negative bacteria and represents a contractile nano-machine that can fire effector proteins directly into neighbouring cells. The primary role assigned to the T6SS is to function as a potent weapon during inter-bacterial competition, delivering antibacterial effectors into rival bacterial cells. However, it has recently emerged that the T6SS can also be used as a powerful weapon against fungal competitors, and the first fungal-specific T6SS effector proteins, Tfe1 and Tfe2, have been identified. These effectors act via distinct mechanisms against a variety of fungal species to cause cell death. Tfe1 intoxication triggers plasma membrane depolarisation, whilst Tfe2 disrupts nutrient uptake and induces autophagy. Based on the frequent coexistence of bacteria and fungi in microbial communities, we propose that T6SS-dependent antifungal activity is likely to be widespread and elicited by a suite of antifungal effectors. Supporting this hypothesis, homologues of Tfe1 and Tfe2 are found in other bacterial species, and a number of T6SS-elaborating species have been demonstrated to interact with fungi. Thus, we envisage that antifungal T6SS will shape many polymicrobial communities, including the human microbiota and disease-causing infections.

## 1. Introduction

Bacteria and fungi are ubiquitous in nature and co-colonise numerous environmental niches. Focussing on the human host, such cross-kingdom interactions are prevalent within the human microbiota, and are commonly associated with biofilms and medically relevant infections [1]. Such interactions may be chemical, physical or occur through alteration of the shared environmental niche, and, importantly, can be synergistic or antagonistic for the species involved. Regarding synergistic interactions, there are several examples of how bacterial and fungal co-infection potentiates host colonisation and virulence [2,3,4], and mixed species biofilms have been shown to create protective environments [5,6]. Conversely, several Gram-negative pathogens, including *Salmonella enterica* serovar Typhimurium [7], *Pseudomonas aeruginosa* [8], and the emerging pathogen *Acinetobacter baumannii* [9], secrete molecules that can kill fungal cells. Perhaps the best characterised is *P. aeruginosa* and its interactions with an important fungal pathogen of humans, *Candida albicans*. *P. aeruginosa* preferentially binds to and forms biofilms on hyphal *C. albicans* cells and kills the fungus through the action of two virulence factors, secreted phospholipase C and redox-active phenazines [8]. *P. aeruginosa* also produces a quorum signalling molecule which inhibits the yeast to hyphal switch [10], an important virulence trait in *C. albicans*.

Whilst antagonistic bacterial-fungal interactions are well recognised, the discovery that a bacterium can directly inject antifungal effectors into fungal cells opens up an exciting new research area underpinning polymicrobial interactions. Recently, we reported that the “antibacterial” Type VI secretion system (T6SS) within the opportunistic pathogen *Serratia marcescens*, is also a potent antifungal weapon [11]. Two dedicated antifungal effectors, Tfe1 and Tfe2, are translocated into fungal cells by the *S. marcescens* T6SS, ultimately resulting in fungal death [11]. Here we give an overview of the T6SS and mechanism of effector delivery before focussing on the identification and mode of action of the newly discovered Tfe1 and Tfe2 antifungal effectors. We also present evidence that T6SS-dependent antifungal activity is likely to be a widespread determinant of microbial community composition, before finishing with the key questions and opportunities for future research afforded by this exciting new area of biology.

## 2. Occurrence of Type VI Secretion Systems

The T6SS is a bacterial nano-weapon that can be used to translocate toxic effector proteins directly into neighbouring cells. It is a versatile system that can be used to deliver antibacterial toxins into rival bacterial cells, representing an important means of inter-bacterial competition, but can also be used to deliver effectors which damage or manipulate host cells, representing direct virulence factors [12]. T6SSs are widely distributed in Gram-negative bacteria. It has been estimated that at least 25% of Gram-negative bacteria contain at least one T6SS, most commonly within the α, β and γ-proteobacteria [13,14]. The majority of such ‘classical’ T6SSs appear to have antibacterial activity, whilst some also, or exclusively, possess anti-host activities. More recently, it has become clear that several other distantly-related families of T6SS also exist, sharing a basic mechanism but with differences in the core machinery. One of these divergent T6SSs occurs in the *Bacteroidetes*, a Phylum including key members of the gut microbiota, and is used for inter-bacterial competition [15,16]. Bacterial species, and indeed individual strains within a species, vary greatly in their complement of T6SSs, from none up to six different T6SSs, whilst the complement of secreted effector proteins is even more variable [12]. In some cases, the same T6SS can be used for two different functions, such as the *Vibrio cholerae* T6SS which is used against both competitor bacteria and the host [17]. In other cases, different T6SSs fulfil distinct roles, for example in *Burkholderia thailandensis*, where T6SS-1 is reported to be exclusively antibacterial, T6SS-5 appears to be exclusively anti-host and T6SS-4 has a distinct role in delivering a manganese-scavenging metallophore protein to the extracellular milieuwhere [18,19]. Regarding potential roles for T6SSs in mediating bacterial-fungal interactions, it is noteworthy that many bacterial species which co-exist with fungi possess T6SSs. Considering bacterial species involved in medically-relevant bacterial-fungal interactions, *P. aeruginosa*, *A. baumannii*, *S. Typhimurium*, *Escherichia coli* and *Burkholderia cenocepacia*, possess well-characterised T6SSs [20,21,22,23,24]. Similarly, many plant-associated bacteria, including plant growth promoting *Rhizobia*, biocontrol organisms such as *Pseudomonas fluorescens* and *Pseudomonas putida*, and plant pathogens including *Agrobacterium tumefaciens* and *Pectobacterium* species, contain T6SSs [25].

## 3. Effector Delivery by the Type VI Secretion System

**Figure 1:**
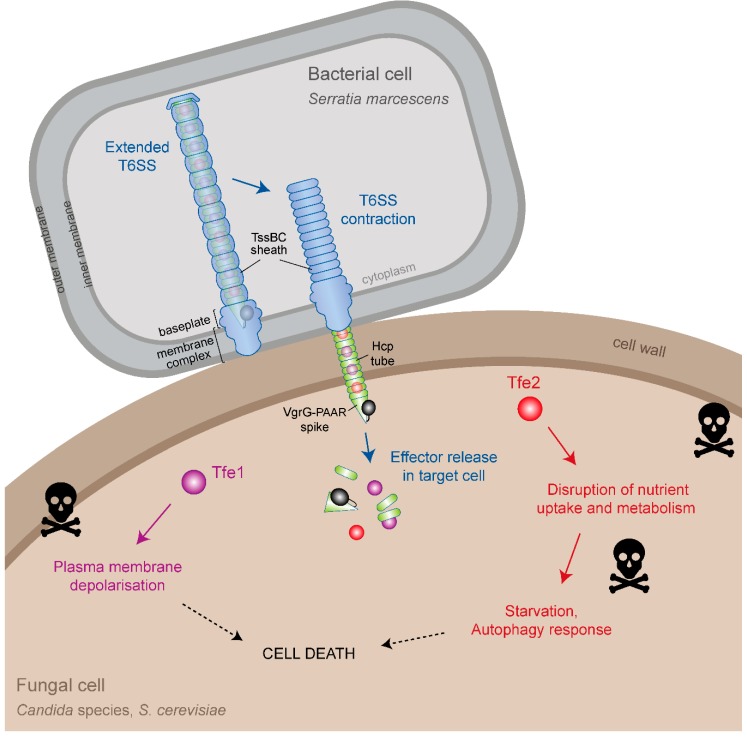
Type VI secretion system-mediated delivery of antifungal effector proteins between *Serratia marcescens* and fungal cells. Schematic representation of the current models for effector delivery by the bacterial Type VI secretion system (T6SS) and the impact of the antifungal effector proteins Tfe1 and Tfe2 on cells of *Candida albicans*, *Candida glabrata* and *Saccharomyces cerevisiae*. In the secreting bacterial cell, contraction of the TssBC sheath propels a cell puncturing structure, decorated with effector proteins, through the membrane-anchored basal complex, out of the bacterial cell and into an adjacent target cell. The cell puncturing structure comprises a tube made of Hcp proteins and a spike made of VgrG and PAAR proteins. Effectors can bind in the lumen of the Hcp tube, as for Tfe1 and Tfe2, or to the outside of the spike (not shown), or they can be present as additional domains fused to spike proteins (example shown in black although antifungal effectors of this kind have yet to be described). Following breach of the target cell due to the mechanical force of the contraction event, effectors are somehow released in the target cell and induce toxicity by distinct mechanisms. In the case of the antifungal effectors, Tfe1 and Tfe2, intoxication leads to plasma membrane depolarisation for Tfe1, whilst it leads to a disruption of nutrient uptake and amino acid metabolism, leading to starvation response and induction of autophagy for Tfe2. Note that the *Serratia marcescens* T6SS also delivers eight antibacterial effector proteins which cause efficient killing of bacterial competitors (not shown).

The T6SS is a large, multiprotein machinery anchored in the inner and outer bacterial membranes. It uses a contraction-based mechanism to propel an arrow-like puncturing device decorated with multiple effector proteins out of the bacterial cell and into neighbouring target cells. In this way, effectors are delivered inside the targeted cell, from where they exert their toxic activities [12]. The mechanism of this machinery, which is related to that of contractile bacteriophage tails, has been reviewed extensively [26,27,28,29] and the current model will be summarised here (Figure 1). The expelled puncturing device comprises a tube made of stacked rings of the Hcp protein, tipped with a sharp spike made of a trimer of VgrG proteins and one PAAR protein. To achieve “firing” of this structure, a membrane complex is assembled across both bacterial membranes, which then serves as a docking site for the cytoplasmic baseplate complex, within which sits the VgrG-PAAR spike. The Hcp tube can then be assembled onto the spike, extending out across the bacterial cytoplasm. A sheath-like structure made up of the TssBC proteins simultaneously assembles around the Hcp tube in an extended, high-energy conformation, with the two structures linked by a “cap” at the distal end. A rapid and powerful sheath contraction event then drives the Hcp-VgrG-PAAR structure through the baseplate and membrane complex, out of the bacterial cell and into a suitably positioned recipient cell, followed by effector release inside the targeted cell. The contracted sheath is disassembled by a dedicated ATPase, TssH, and further rounds of T6SS assembly and firing can occur. 

In order to achieve effector delivery, effectors associate with the Hcp-VgrG-PAAR structure in a number of ways [12]. They can interact non-covalently with the inside of the Hcp tube or with the outside of the VgrG-PAAR spike. Alternatively, VgrG, Hcp and PAAR proteins may possess additional effector-containing domains, normally at their C-termini. To accommodate multiple different effector proteins, T6SSs typically contain multiple copies of Hcp, VgrG and/or PAAR proteins and often possess effector-specific chaperones to aid loading of the effector onto the machinery. The T6SS appears to be a very flexible delivery machine, able to deliver effectors of a variety of sizes, structures and functions. These range from antibacterial effectors with cell wall hydrolase, phospholipase and nuclease activities, through metal scavenging proteins, to anti-host effectors with actin modification, inflammasome modulation and membrane fusion functions [12,30,31].

## 4. Identification of T6SS Antifungal Effectors

The first indications that the T6SS may play a role in bacterial-fungal interactions were reported for plant-associated bacteria, which frequently share their habitat with symbiotic or disease-causing fungi [32,33]. More specifically, the plant-associated Pseudomonad, *P. fluorescens* Pf29Arp, which has been shown to protect wheat roots from the pathogenic fungus *Gaeumannomyces graminis var. tritici*, exhibited increased expression of T6SS genes if cultured on fungus-infected roots compared with healthy roots [33,34]. In addition, direct T6SS-dependent activity against both bacterial and fungal competitors was reported for the pathogenic phytobacterium *Pseudomonas syringae* pv. tomato DC3000 [35]. However, in both cases, no antifungal T6SS effectors were identified. In contrast, effector-based antifungal activity was reported for the T6-secreted protein Tse2 of *P. aeruginosa* when overexpressed ectopically in the model yeast *S. cerevisiae* [36,37]. Similarly, transfection of HeLa cells with Tse2 led to cell-rounding. However in physiologically more relevant co-culture experiments, when Tse2 would be delivered by the T6SS, Tse2 toxicity was restricted only to other bacteria [37]. Subsequent structural analysis of Tse2 revealed identity with ADP-ribosyltransferase toxins [38], suggesting general cytotoxic activity towards an evolutionary conserved target rather than being an effector deployed against fungal cells. 

Identification of the first fungal-specific T6-secreted effectors was recently reported for the model strain, *Serratia marcescens* Db10 [11]. *S. marcescens* possesses a single potent antibacterial T6SS, which has been shown to deliver at least eight distinct antibacterial effectors, and is post-translationally regulated to fire in an offensive manner [39]. Co-culturing *S. marcescens* with the model yeast *S. cerevisiae*, or the opportunistic pathogenic fungi *C. albicans* and *C. glabrata*, resulted in T6SS-dependent inhibition of fungal growth. This effect was dependent on cell-to-cell contact, dispelling doubts about whether the T6SS could breach the thick and rigid fungal cell wall.

To identify the effectors responsible for antifungal activity, initial experiments focussed on T6-secreted proteins which had previously been identified in a proteomics screen comparing the secretome of wild type *S. marcescens* with that of a T6SS-inactive mutant [40]. Of these, only one, Ssp3, exhibited fungicidal activity against *C. albicans*. Interestingly, Ssp3 was initially classified as an antibacterial toxin, since overexpression in *E. coli* produced modest toxicity, which was alleviated upon co-expression of a small open reading frame situated directly upstream of the toxin [40]. However, deletion of this immunity gene, *sip3*, in *S. marcescens* did not affect viability, and no inhibition of the *Δsip3* mutant upon co-culture with strains able to perform T6SS-mediated delivery of Ssp3 was observed, indicating that Ssp3 is not an antibacterial effector under such conditions [11]. This is in contrast with true T6SS antibacterial effectors, where mutants lacking the cognate immunity protein are non-viable due to self-killing and are susceptible to delivery of the cognate effector upon co-culture with a wild type strain. To reflect its antifungal rather than antibacterial activity and its identification as the first T6SS-secreted antifungal effector protein, Ssp3 was renamed Tfe1. However, deletion of *tfe1* in *S. marcescens* did not reduce antifungal activity against *S. cerevisiae* or *C. glabrata* in co-culture experiments, suggesting the existence of additional antifungal effectors. To identify such effectors, a second proteomics approach was employed to capture the cellular proteome, rather than the secretome, with the rationale that a T6SS-inactive mutant would retain potential effector proteins inside the cell. Analysis of the cellular proteome of a Δ*tssE* mutant compared with wild type *S. marcescens* revealed twelve proteins that displayed increased abundance in the T6SS-inactive mutant. Nine were proteins already known to be secreted by the T6SS, including components of the puncturing structure and Tfe1, whilst the remaining three proteins were unlikely to be antibacterial effectors since they were not encoded next to potential cognate immunity proteins. Subsequently, mutational analysis identified one of these three candidates as the second antifungal effector, Tfe2 [11]. Notably, whilst Tfe2 failed to exhibit antibacterial activity, loss of Tfe2 virtually abolished *S. marcescens* antifungal activity against *S. cerevisiae* and *C. glabrata*. Thus the “antibacterial” *S. marcescens* T6SS is also a potent antifungal weapon, able to kill *S. cerevisiae* and *Candida* spp. by delivering two dedicated antifungal effectors Tfe1 and Tfe2.

Given rapid recent advances in the T6SS field, relating to both its structure and secretion mechanism [41,42,43,44] and the diversity of secreted proteins [12], it is perhaps surprising that antifungal-specific effectors have been identified only recently [11]. This is likely due to the fact that the strategies used to discover novel effectors have been based on the assumption that T6SSs are primarily employed as weapons in interbacterial warfare or else as classical virulence factors against higher eukaryotes. Therefore, effector identification strategies have often relied on their presumed antibacterial activity and the presence of cognate immunity proteins. For example, using unbiased proteomics approaches, only those T6-secreted proteins harbouring a potential immunity protein were considered candidate T6SS effector proteins, with confirmation being based on their ability to kill a non-immune sibling strain [37,40]. Similarly, effector identification via random transposon mutagenesis and deep sequencing (Tn-seq) in wild type and T6SS inactive mutant backgrounds relied on the identification of immunity proteins as being essential in the presence of a functional T6SS [45]. However, by using such strategies antifungal effectors would be missed. Indeed, no immunity protein is associated with Tfe2, consistent with the premise that bacteria deploying fungal-specific toxins would not require cognate immunity proteins for protection. Furthermore, hypothesis-driven in silico discovery of T6SS effectors, based on domain and homology screens (e.g., peptidoglycan-degrading enzymes [46], lipases [47] and MIX-motif containing proteins [48]), are led by the prior knowledge of previously identified effectors and thus are likely to miss novel fungal-specific effector proteins. Alternatively, effectors are often located within T6SS gene clusters or distant loci encoding additional Hcp, VgrG and/or PAAR proteins, and so candidate effectors can be identified by their genetic context. Similarly, candidate effector domains fused with VgrG or PAAR proteins are readily identifiable through bioinformatic analysis. However, these approaches will miss effectors located in genomic regions otherwise unrelated to T6SS, such as Tfe1 and Tfe2, and to-date have again typically relied on identification of immunity proteins and/or demonstration of antibacterial activity as validation as bona fide T6SS substrates.

It is also important to note that antifungal effectors (like antibacterial or anti-host effectors) may be delivered by T6SSs that are silent under laboratory conditions, thus hindering their identification. Knowledge about signalling pathways and regulatory mechanisms, in combination with genetic tools to generate constitutively active T6SSs, may prove instrumental in the discovery of future T6SS effectors. This has been exemplified by deletion of the gene encoding for the sensor kinase RetS in *P. aeruginosa* or the quorum-sensing master regulator OpaR in *V. parahaemolyticus*, both rendering the respective T6SSs constitutively active [49,50]. Similarly, interfering with the phosphorylation status of the scaffolding protein Fha1 by deleting the Ser-Thr phosphatase PppA in *P. aeruginosa* locks the T6SS in its active state, which allowed for identification of T6-secreted proteins under standard laboratory conditions [51]. Future strategies to study such silent T6SSs will likely require a combination of genetics tools together with performing co-culture experiments under physiologically relevant conditions. In this regard it is notable that the laboratory silent T6SSs of *V. cholerae* O1 serogroup strains C6706 and N16961 are stimulated by intestinal factors, namely, mucins and bile salts [52].

## 5. Mode of Action of Tfe1 and Tfe2

Tfe1 and Tfe2 are small proteins comprising 183 and 226 amino acids with predicted molecular masses of 20 and 26 kDa, respectively. No discernible conserved domains or predicted functions could be identified for either protein, using standard bioinformatics tools. Once inside the fungal cell, Tfe1 and Tfe2 trigger distinct morphological responses, with Tfe1 causing cell distortion and lysis and Tfe2 giving rise to granular structures [11].

Using the voltage-dependent dye DiBAC_4_(3), which can only enter depolarised cells, Tfe1 was found to trigger a loss of plasma membrane potential [11]. Only a small fraction of Tfe1-intoxicated cells co-stained with propidium iodide (PI), an indicator for loss of membrane integrity. This indicates that Tfe1 causes loss of plasma membrane potential without forming pores, similar to the action of the membrane depolarising antifungal drug amphotericin B, but in contrast to the pore-forming peptide mellitin. This effect was seen upon direct co-culture of *C. albicans* with wild-type *S. marcescens* but not the *Δtfe1* mutant, as well as upon ectopic expression of Tfe1 in *S. cerevisiae*, suggesting Tfe1 is targeting a conserved fungal pathway. Interestingly, although the fungicidal action of Tfe1 was confirmed by decreased survival rates of *C. albicans* in co-culture settings, both *S. cerevisiae* and *C. glabrata* are much more resistant to Tfe1 action. As the latter are phylogenetically more closely related to each other than to *C. albicans*, the observed species specificity of Tfe1 might be related to a divergence in the targeted pathway between those two clades.

The precise mechanism of Tfe1-elicited membrane depolarisation is unknown. In *S. cerevisiae*, membrane potential is controlled mainly by the regulation of proton and potassium cation fluxes, with the main regulator being the extensively studied and well-characterised Pma1 H^+^-ATPase [53]. This protein pumps protons out of the cytosol to regulate intracellular pH and creates a proton electrochemical gradient across the plasma membrane. If Tfe1 functions to inhibit Pma1, this would block H^+^ efflux from the cell resulting in loss of membrane potential, as has been recently reported for several antifungal compounds acting on Pma1 [54]. Alternatively, Tfe1 may impact on the activity of K^+^ transporters to trigger membrane depolarisation [55], or via a mechanism akin to the antifungal amphotericin B or, indeed, via a novel mechanism.

The second antifungal effector, Tfe2, was the most potent toxin against *S. cerevisiae* and *C. glabrata*, whilst displaying approximately equal activity as Tfe1 against *C. albicans* [11]. Whilst PI staining of *C. glabrata* co-cultured with *S. marcescens* suggested Tfe2-dependent killing of the fungus, Tfe2-intoxication of both *S. cerevisiae* and *C. albicans* was fungistatic, with reduced long-term survival. These data suggest that Tfe2 targets a conserved pathway, but with distinct fungi exhibiting varying levels of susceptibility. To determine the impact of Tfe2 intoxication, an “in competition” proteomics experiment was performed to identify *C. albicans* proteins differentially affected upon co-culture of *C. albicans* with wild type *S. marcescens* compared to co-culture with mutants of *S. marcescens* unable to deliver Tfe2 [11]. This revealed a Tfe2-dependent reduction in nutrient transporters, including the sole sulphate transporter Sul2, and the amino acid permeases Gap1, Gap2, Can1 and Can2. Moreover, the entire sulphate assimilation pathway was downregulated upon Tfe2 intoxication. In contrast, Tfe2 intoxication stimulated increased levels of the Gcn4 transcriptional activator which mediates the general amino acid control (GAAC) response following amino acid starvation [56,57], and the autophagy-activating Ser/Thr kinase Atg1, which is subject to regulation by the nutrient-sensing TOR signalling pathway [58]. These data strongly suggested interference of Tfe2 with nutrient sensing or uptake pathways. Although no direct target was identified, certain hypotheses were considered. First, decrease in transporter levels could occur via transcriptional downregulation. However, increased transcript abundance of transporters or their transcriptional regulators was observed upon exposure to Tfe2, likely as a compensatory mechanism. Alternatively, Tfe2-mediated decrease in transport proteins could be triggered by nutrient-binding induced endocytosis, but the probability of this scenario is low due to invariant media composition. Perhaps Tfe2 functions to interfere with protein synthesis or vesicle trafficking/premature vacuolar targeting which could underlie the decrease in nutrient transporters, which in turn would elicit nutrient starvation response pathways. Whatever the mechanism, Tfe2-mediated depletion of nutrient transporters at the post-transcriptional level leads to amino acid imbalance resulting in induction of autophagy. Which, if any, of these Tfe2-dependent effects is responsible for fungal death remains to be determined. However, it is noteworthy that deletion of the basic amino acid transporter Can1 and its paralog Alp1, which are down-regulated by Tfe2, give a lethal phenotype in *S. cerevisiae* [59]. In addition, Tfe2 increases levels of the Gcn4 transcriptional activator, and previous studies have revealed that Gcn4 over-expression stunts growth [60].

The *S. marcescens* T6SS, and by implication Tfe1 and Tfe2, do not appear to act against higher eukaryotic cells as strains lacking a functional T6SS show no loss of virulence [11,39]. Furthermore, Tfe1 and Tfe2, which trigger plasma membrane depolarisation and impaired nutrient uptake, respectively, seemingly display a different mode-of action to T6SS-delivered anti-host effectors reported to date. Such anti-host effectors target diverse processes encountered by pathogens during infection to allow immune evasion [30]. Several manipulate the host cytoskeleton, via the generation of toxic actin oligomers in *V. cholerae* V52 [61], by inhibiting F-actin formation in *A. hydrophila* [62], or by activation of the Pyrin inflammasome via Rho GTPase deaminase activity in *B. cenocepacia* [63]. Other anti-host effectors include those able to modulate microtubule-mediated bacterial internalisation by binding to tubulin or Akt, those able to promote phagosomal escape through PI(3)-kinase or membrane fusion activities, and those able to counter inflammasome activation or host ROS, as reviewed in [12]. Based on the recent findings that the T6SS can target fungal competitors [11], it will be interesting to ask whether previously designated anti-host effectors are also active against fungal cells.

## 6. Prevalence of T6SS-Dependent Antifungal Activity

**Figure 2:**
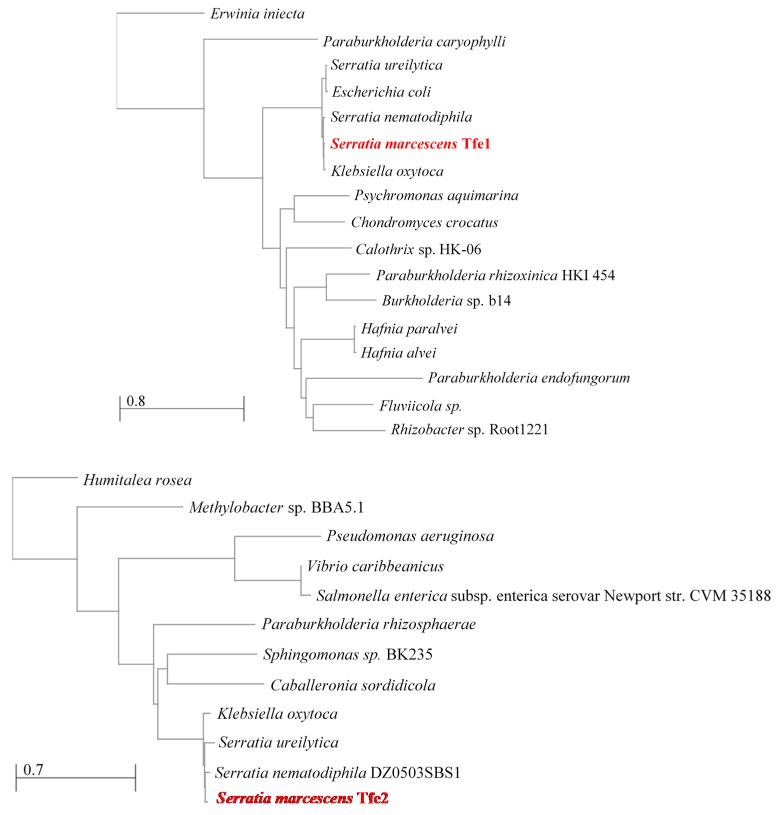
Neighbour joining trees depicting relatedness of Tfe1 and Tfe2 found in other bacterial species. Specific strains are indicated when known.

Due to the frequent co-existence of T6SS-containing bacteria and fungi in microbial communities, it is likely that T6SS-mediated antifungal activity is widespread. For example, multiple T6SSs are prevalent among major constituents of the gut microbiota, the *Bacteroidetes* [64], which comprise approximately half of the colonic bacteria within the human population. Furthermore, 73% of 143 analysed plant-associated bacteria of the Proteobacterium phylum possess at least 1 and up to 5 distinct T6SS clusters [25]. Such T6SS-containing plant and human associated bacteria are intimately associated with fungi, supporting the concept that T6SS antifungal activity is not restricted to *S. marcescens*. Indeed, our analysis has revealed that Tfe1 and Tfe2 homologues are found in a number of bacterial species. Conducting neighbour-joining tree analyses of Tfe1 and Tfe2 using the non-redundant sequence database of NCBI depicted that both effectors were restricted to Gram-negative bacteria but not exclusive to the *S. marcescens* clade (Figure 2). Indeed, homologues are present in a variety of proteobacteria, including closely related *Enterobacterales*, but also more distantly related species like *Burkholderiales*, *Pseudomonadales* and *Vibrionales*, representing both human and plant-associated pathogens and environmental strains. All species possessing a Tfe1 or Tfe2 homologue also contain at least a potential *vgrG* gene in their genome which is indicative of a functional T6SS. However, whether all strains within a particular species contain Tfe1 or Tfe2, and the genes necessary for an active T6SS machinery, requires further analysis. We also posit that Tfe1 and Tfe2 will be representatives of an array of novel T6-secreted antifungal effectors. In this regard it is relevant that Tfe1 and Tfe2 are not present in the plant associated strains *P. fluorescens* Pf29Arp and *P. syringae* pv. tomato DC3000, in which anti-fungal T6SS activity was first described [33,34,35]. Furthermore, as detailed above, antifungal effectors may have been missed in many other studies due to the standard identification criteria being based on antibacterial activity (which Tfe1 and Tfe2 do not display). These include proteins omitted due to the lack of antibacterial activity upon deletion of the respective cognate immunity protein, or the absence altogether of potential immunity candidates (e.g., VP1390 in *V. haemolyticus* [48]).

## 7. Roles for Antifungal T6SSs in Polymicrobial Communities?

The majority of studies on antibacterial T6SSs have concentrated on in vitro competition experiments which has driven the rapid advancement of our molecular understanding of the T6SS. Although fruitful, such approaches give limited insight into the functionality of T6SSs in natural polymicrobial communities. However, recent studies have revealed that a range of bacteria utilise their T6SS to confer a competitive advantage within the polymicrobial community of the gut. This was demonstrated in *S.* Typhimurium where the T6SS encoded within Salmonella pathogenicity island 6 was shown to promote successful colonisation of the mouse gut [22]. Similarly, *Shigella sonnei*, but not *Shigella flexneri*, contains a T6SS that allows it to outcompete *S. flexneri* and *E. coli* in the mouse gut, and it is suggested that this T6SS-conferred competitive advantage may contribute to the global rise in *S. sonnei* incidence [65]. The *V. cholerae* T6SS not only antagonises commensal *E. coli* species, but also functions to enhance the virulence of this enteric pathogen [66]. Specifically, T6SS-mediated killing of *E. coli* commensals both stimulated *V. cholerae* virulence gene expression and intensified innate immune responses [66]. In addition to these enteric pathogens, T6SS are employed by Bacteroidales species. Three different genetic architectures (GA) of T6SS are prevalent; GA1 and GA2 are located on mobile genetic elements and are readily transferred and shared amongst numerous Bacteroidales spp, whereas GA3 is restricted to *Bacteroides fragilis* [64]. *B. fragilis* utilises its GA3 T6SS to antagonise human gut Bacteroidales species, providing a competitive advantage in murine co-colonisation experiments [67].

*S. marcescens*, which has a potent antifungal T6SS, is emerging as an important opportunistic pathogen [68], and co-colonisation of this bacterium with commensal *Candida* species in dysbiotic human gut communities has been reported [69]. In addition, co-infection experiments with other medically-relevant, T6SS yielding, bacterial species including *A. baumannii* and *S.* Typhimurium have been shown to decrease *C. albicans* virulence in the intestine of the model invertebrate host *Caenorhabditis elegans* [7,9]. Collectively, these findings support the concept that antifungal T6SS may play a vital role in modulating the fungal component of the gut microbiota. This may have medical relevance, as growing evidence indicates that the mycobiota, like the bacterial microbiota, plays important roles in immune homeostasis and health [70].

## 8. Outstanding Questions

The recent discovery that bacteria can use the T6SS to interact with fungal cells raises many exciting questions and opportunities for future research. Given the ubiquitous nature of bacterial and fungal cells, and their frequent co-occurrence in polymicrobial communities, we speculate that T6SS-mediated bacterial-fungal interactions will turn out to be important in a multitude of biological contexts. However the extent, mechanisms and importance of these interactions remains to be established; with key questions including:

- How many bacteria with T6SSs can use these systems against fungal cells? It will also be interesting to see if T6SSs dedicated to antifungal activity exist, perhaps regulated in response to fungal cues, or if antifungal and antibacterial activity always co-occurs.
- How broad is fungal susceptibility to T6SS action? It remains to be seen whether other species, genera and phyla can be targeted by bacterial T6SSs and whether particular T6SSs and/or effector proteins are specific for different types of fungi. For example, it is unknown if true filamentous fungi are susceptible to T6SS action. It is tempting to speculate that the composition of the fungal cell wall may play a critical role in determining the efficacy of T6SS attacks.
- What are the precise modes of action of Tfe1 and Tfe2, and yet-to-be-identified T6SS antifungal effectors, and how many toxin molecules are required to cause fungal death? It will be very interesting to discover the range of activities such effectors might have and to determine whether any effectors that act against fungal cells also act against higher eukaryotic host organisms. Indeed we speculate that some of the effectors reported to act against host cells might have originally been acquired to act against fungal competitors, as targets such as actin are conserved throughout the eukaryotic kingdom.
- Can bacteria also use T6SSs to deliver effector proteins that promote positive, mutualistic interactions between bacterial and fungal cells, rather than being solely antagonistic?
- What is the significance of T6SS-mediated bacterial-fungal interactions in “real-life” polymicrobial communities? We look forward to learning how these interactions can change the balance between health and disease, influence the gut microbiota or define the composition of environmentally-important communities.
